# The Entanglement Between Tangible and Intangible Factors in Shaping Hadiya Migration Aspirations to South Africa

**DOI:** 10.1177/01979183241226635

**Published:** 2024-02-06

**Authors:** Dereje Feyissa Dori, Jessica Hagen-Zanker, Caterina Mazzilli

**Affiliations:** 37602 College of Law and Governance, Addis Ababa University, Addis Ababa, Ethiopia; 4837 ODI, London, UK

**Keywords:** Migration decision-making, subjective and intangible factors, Hadiya migration to South Africa

## Abstract

This article expands scholarly knowledge on migration decision-making drawing on the case of Hadiya (Southern Ethiopia) migration to South Africa. We propose a conceptual framework where intangible factors (religious beliefs, imaginations, norms, and emotions, and feelings) are placed at the core of decision-making, alongside more tangible factors, such as livelihood opportunities. Even though we differentiate between intangible and tangible, we reject any opposition between the two, arguing that they inform and reinforce each other. The Hadiya—South Africa migration corridor emerged from a foundational evangelical Christian prophecy in 2001, which coincided with other events in the Hadiya zone, such as increasing pressure on farmlands, the politicization of internal migration, and more liberal migration policies. Drawing on extensive qualitative data, we focus on the relevance of intangible factors in Hadiya migration to South Africa, bringing to the fore an understudied aspect of decision-making. Showing the centrality of such aspects in Hadiya respondent's life stories, we argue that only by looking at the interplay of intangible and tangible factors we can reach a better understanding of the complex dynamic of migration decision-making.

## Introduction

This article expands scholarly knowledge on intangible factors’ relevance in migration decision-making and on their interplay with tangible factors. It builds on a growing body of literature on the role of subjective and intangible factors in migration decision-making (reviewed in Hagen-Zanker, Mazzilli, and Hennessey 2023) to examine the spiritual and emotional—and to a lesser extent imaginative—dimensions embedded in the Ethiopia-South Africa migration corridor. The article centers around the life stories of Hadiya people of Southern Ethiopia, for whom migration to South Africa has become the main future-making project in the last two decades.

Since an evangelical Christian prophecy given by pastor Peter Younger in Hosana in 2001 revealed the opening of “a route to the South,” which “would bring prosperity to Hadiya,” migration from this area of Ethiopia to South Africa has become a constant, imbued with spirituality and driven by emotions such as admiration and sense of adventure, but also peer competition and emulative jealousy. This migration continues to this day, despite dangers during the long journey and at the destination. Risks during the journey include detention, hazardous means of transport, abandonment by brokers, and/or death from starvation. Such incidents happen frequently: for instance, in December 2022, the bodies of 29 Ethiopian migrants were found in Zambia, suspected to have died of hunger and exhaustion (DW 2022). Obstacles do not end once migrants reach South Africa: there, they are also subjected to growing xenophobic attacks and robberies, as well as to a rising homicide rate among migrants generated by competition over business turfs.

Our research shows that spirituality is crucial for Hadiya's sense-making of their life story and for their own moral support toward the goal of establishing a successful life in South Africa. In addition, feelings of admiration for those who have already left, mixed with peer competition and emulative jealousy, push increasingly younger Hadiya to leave for South Africa. And yet, this story cannot be told exclusively through the lens of intangible factors: Hadiya migrants in South Africa experience hard times but *do* have access to economic and entrepreneurial opportunities in one of the continent's richest countries that they would not have had access to in Ethiopia. It is hard to imagine that this migration would have taken place without the economic prosperity associated with it.

The determination with which prospective migrants in Hadiya continue to invest in the migration project, despite all of its risks, calls for a conceptual framing, which is the first contribution of our article. The way we frame migration decision-making builds on recent advancements in the literature on decision-making, which has moved away from old approaches such as the push/pull narratives or the functionalism/historical-structuralism approaches. Our framing shows that intangible and tangible factors are different but not opposed, rather informing each other. In this way, we also offer a solution to de Haas’s (2021) puzzle on how to tell apart intrinsic and instrumental migration aspirations. In our view, the more we oppose one another, the more they in fact look entangled. Therefore, we bring tangible and intangible factors closer together, demonstrating not only that both matter for Hadiya migrating to South Africa, but also that they mutually reinforce each other's relevance. We also show how Hadiya migration to South Africa has over time morphed from being triggered by more intangible to more tangible factors and from being perceived as a redemption project for the Hadiya people to a personal realization journey. This point demonstrated that migration projects do not remain identical but keep changing as individuals are embedded into big ever-changing structures.

Presenting this case study through the above conceptual framework, our overall contribution to the migration literature is to make the complexity of the migration process more intelligible. The article proceeds as follows: we first elaborate on our conceptual framing, placing subjective and intangible factors within the migration decision-making literature. We then present our data and methodological approach. After giving an overview of the evolution of Hadiya migration to South Africa and key tangible factors, our analysis centers around four key categories of intangible factors: beliefs, imaginations, social norms, and emotions/feelings. Our concluding section shows how closely intertwined these intangible factors are with the economic drivers with various degrees and modes of signification.

## Conceptual Framing

### The Transformation of the Literature Over Time

Broadly speaking, much of the literature on migration decision-making could long be divided into two main approaches, functionalism, and historical-structuralism,^1^ both centering around the question of why some people move while others stay put. Functionalism conceptualizes migration as a rational choice that an individual makes to secure greater income and wider opportunities, after evaluating socioeconomic costs and benefits (de Haas 2021). Best known are Harris and Todaro's (1970) neoclassical migration theory and the “push-pull” model (Lee 1966), which described push and pull factors as respectively unfavorable and attractive elements that become reasons to either leave the country of origin or move to a certain country of destination. Historical-structuralism, on the other hand, focuses on structures rather than individual agency, depicting migration as the result of socioeconomic inequalities between individuals and states (see, e.g., Wallerstein 1974 and Piore 1979). In fact, this approach conceives migration as an irrational process that migrants are drawn into because of distorted information or an exploitative macrostructure that entraps them (de Haas 2021).

Functionalism has the merit of acknowledging migrants’ agency but provides an oversimplified version of the messiness of human nature and of the dynamics at play in the world. Push-pull factors are a case in point since, despite rich evidence that migration decision-making is much more nuanced (as discussed below), they remain a well-established framing for the reasons behind migration both at the political and scholarly level (Brzozowski and Coniglio 2021; Ferwenda and Gest 2021; Hager 2021)—perhaps because of this model's simplicity. Like Massey et al. (1993), Arango (2000), and Carling (2014) have pointed out, functionalism describes human beings as mostly rational actors, but also locates them in an environment where all choices are equally possible, individuals have access to perfect information, and the only structure they are embedded in is the market. Feyissa (2022) argues that this preoccupation with economic factors “has come with a reality cost, to the extent that the noneconomic factors in migration processes are not given the attention they deserve” (p. 36).

While historical-structuralism paid attention to the power structures individuals are embedded in—be them economic, political, class, or gender, it has as such conceptualized migrants as victims of these forces, leaving no space for agency. For instance, it does not explain how migrants retain agency even under difficult conditions, or why individuals facing the same structural constraints react to them in different ways.

Although these paradigms continue to exert a considerable influence on current scholarship and policy-making, over the last two decades research on migration decision-making has expanded greatly, gradually acknowledging the complexity of this dynamic, and moving from conceiving decision-making as a one-time decision of whether (or not) to migrate onto analyzing migration mode, journeys, encounters, as well as destinations (Crawley and Hagen-Zanker 2019; Gladkova and Mazzucato 2017; Hagen-Zanker and Mallett 2016) and calling out the mobility bias in the literature that has resulted from the “obsession” with the drivers of migration (Schewel 2020).

### Turning the Spotlight on the Invisible Factors on Migration

Not only have studies on decision-making multiplied, but scholars have also proposed new paradigms to capture this advance in understanding. For instance, de Haas (2021) proposed a framework that “conceptualises migration as a function of aspirations and capabilities to migrate within given sets of perceived geographical opportunity structures” (p. 1), as such bridging functional and historical structural approaches. In fact, there is now a rich theoretical and empirical body of work around the aspirations-capability framework (Carling and Schewel 2018; Schewel 2020; Wyngaarden et al. 2023). However, this article takes a step forward in a different direction. Inspired by hundreds of studies on intangible factors (Hagen-Zanker, Mazzilli, and Hennessey 2023), we demonstrate that intangible and tangible factors are equally powerful but also deeply entangled in the life stories of Hadiya migrating to South Africa.

We define unobservable or intangible factors as those inside a person's mind, the ways in which they see the world and their place within it, which cannot be systematically measured or observed by others as they do not univocally correspond to a change in material conditions. Tangible factors on the other hand, such as wage differentials or migration policies, while also not always clearly visible or available to all, can in theory be observed and measured. Even conceptually more “fuzzy” terms like social class—understood as disposal of different forms of capital, can be defined and observed in terms of their relationship to migration—for instance by identifying who can move and from/to where (Van Hear 2014) or gauging the improvements/worsening of someone's socioeconomic situation.

Following Hagen-Zanker, Mazzilli, and Hennessey's (2023) classification, we divide subjective and intangible factors into four categories: imagination, personality traits, emotions and feelings, and beliefs and values. Imagination refers to those mental processes—also called “mental journeys” (Cangià and Zittoun 2020) that allow a person to visualize their life in a different place and their own future self in there. Personality traits and their connection to migration have been largely elaborated on personality classification models developed at the end of the 1980s, with more recent studies focusing on traits other than those originally included in the model,^2^ such as for instance adaptability and patience (Goldbach and Schlüter 2018).

Emotions and beliefs have been comparatively less explored in the literature. Yet, there are several articles showing the connections between migration and feelings of, for instance, entrapment, jealousy, and frustration for someone's life situation (Kalir 2005; Belloni 2019). Although it is normal to assume that emotions and feelings are deeply subjective, they are also mediated by the environment individuals live in. A case in point, Zagaria (2019) explains how social expectations around young Tunisian men mediate feelings such as jealousy, which commonly lead to migration. This dynamic emerged clearly also from our research on Hadiya migration, as driven by a double-faced mix of admiration and emulative jealousy for those who have left and managed to achieve success in South Africa.

Spirituality, a key factor we observe in this research, falls within the beliefs and values category, although the category includes many more elements such as political beliefs or attitudes toward gender equality (Docquier, Tansel, and Turati 2020; Etling, Backeberg, and Tholen 2020). Research has highlighted the importance of religion (and, broadly, spirituality) in producing hope and trust in the existence of a divine script (Hoffman, Marsiglia, and Ayers 2015; Bredeloup 2019). This not only helps migrants to overcome hard times at various moments of their migration journey but also gives them a sense of legitimacy and purpose—as emerges from the accounts of Hadiya migrants. In addition, spirituality can influence imagination: framing South Africa through the prism of Pastor Peter Youngren's prophecy (described below), Hadiya see their destination as a “promised land” capable of bringing prosperity to themselves, their family, and hometown.

**Figure 1.**
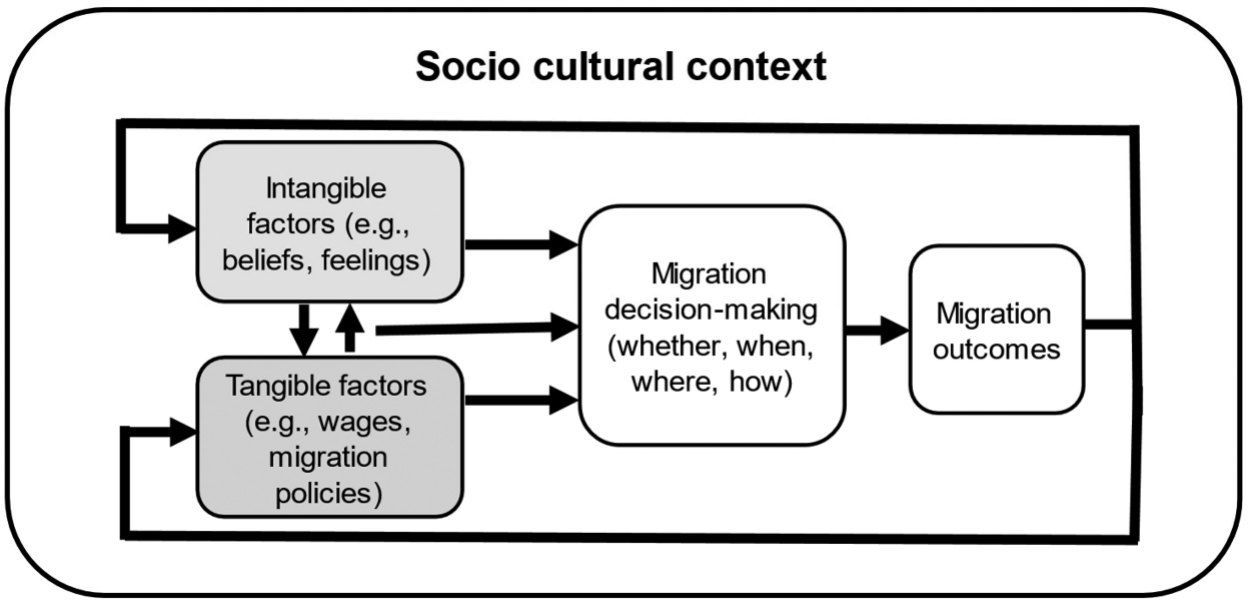
A framework linking intangible to tangible factors.

In this article, we place these invisible factors in a migration decision-making framework alongside tangible factors, arguing that both affect migration decision-making, which in turn results in migration outcomes (which can include immobility) (Figure 1). For example, failed migration attempts can shape risk preferences (Bocquèho et al. 2018), triggering emotions such as shame that spur further re-migration (Carling and Haugen 2021) or generating different migration imaginaries. Moreover, the interplay between tangible and intangible factors can also affect decision-making. Finally, as historical-structuralist models taught us, these decision-making factors and processes are formed within specific sociocultural contexts, for instance around the acceptability of irregular migration (Ryo 2013; Schwartz et al. 2016) and imaginations of migration are both embedded in socioeconomic structures and culturally transmitted (Thorsen 2010).

Having laid out our conceptual framing for the study, we now move on to the empirical analysis.

### Data and Methodological Approach

The empirical base of this contribution comes from the ethnographic study conducted between 2019 and 2022 by the Migration for Development and Equality (MIDEQ) research project. It involved, among other methodologies, the collection of the life stories of 60 migrants who returned to Ethiopia and five who still reside in South Africa. Moreover, the research team conducted Key Informant Interviews and In-Depth Interviews (IDIs) with migrant families, aspiring migrants, church leaders, and officials from government institutions. The research participants were selected partly through purposive sampling and partly on the basis of availability and interest to participate. They took place in Hosanna, Jajura, Shashogo, and Fonqo, all part of the Hadiya administrative zone.

Initially, the Ethiopia-to-South Africa corridor was not part of MIDEQ's work on decision-making, rather focusing on resource flows, children staying back, and income inequality. Issues related to decision-making came up organically as the research participants described their experience of migration, frequently starting with why and how they migrated. The theme of spirituality in relation to migration invariably came up as a strong factor in decision-making across the majority of the 100 interviews that the team conducted.^3^ As such, the team decided to explore the role of subjective factors in this migration in more depth.

All the 60 informants whose stories are included in this article are men, as the corridor is very much gendered. Hadiya men mostly migrate to South Africa, while Hadiya women migrate to Gulf countries. Different age groups are represented in the sample: 75 percent of the selected participants were between the ages of 25 and 35; 20 percent were between 36 and 50, and 5 percent were above 50. More than 90 percent of the research participants are evangelical Christians, reflecting Hadiya's religious demography. In addition, 90 percent of the participants had lived in South Africa for 10 years at the time of the interview, 7 percent had lived there for a period between 11 and 15 years, and the remaining 3 percent for less than 10 years.

Still, to mitigate the potential pitfalls of retrospective narratives and what Jerolmack and Khan (2014) call the “attitudinal fallacy”—that is, the conflation between self-reported oral accounts with behavior, the emerging patterns from life histories were triangulated with other data, such as IDIs with aspiring migrants and others. There was consistent agreement across the board on the influence of spirituality in Hadiya migration, even by those interviewees who stated to be skeptical about the content of the founding prophecy. Ethnographic observation of Hadiya's everyday forms of spirituality such as sermons, preaching, and gospel songs with migration content was used to generate additional insight into Hadiya's self-understanding as vanguards of evangelical Christianity and receptivity to prophetic messages.

Most of the interviews were conducted in the Amharic language, the national language in Ethiopia and the mother tongue of the research team—which most Hadiya are conversant with. Some of the interviews, especially in rural Hadiya, were conducted in Hadiyissa by a Hadiya member of the team. The interviews were then transcribed and translated into English and made accessible to the wider MIDEQ Research Hub. Observing the ethical protocol, the names of research participants are anonymized, and potentially sensitive issues are protected, ensuring no harm is done not only to the research participants but also to the wider Hadiya society.

The data extracted from the life histories were analyzed with reference to the four categories of subjective factors categorized by Hagen-Zanker, Mazzilli, and Hennessey (2023): imagination, personality traits, emotions and feelings, and beliefs and values. Spirituality (beliefs), emotions, and social norms (values) emerged as most relevant for this context, which in turn informed the presentation of findings.

### Hadiya Migration to South Africa

As one of the strongest economies on the continent, South Africa is among the major destination countries for migrants moving within Africa. Close to three million migrants resided in South Africa in 2020 (UNDESA 2020). Ethiopians are among the most significant of these migrant populations, with estimates counting as many as 250,000 (Cooper and Esser 2018). Most of these Ethiopian migrants come from Southern Ethiopia, particularly from the Hadiya-Kembatta area.

Most Ethiopian migrants in South Africa have settled in Jeppe, the Ethiopian commercial enclave in Johannesburg, and in the nearby townships (Zack and Estifanos 2016). Most are undocumented or only have permission for a temporary stay, with few managing to attain refugee status with proper documents. Nearly all Hadiya migrants are self-employed entrepreneurs working in informal trade, initially engaged in door-to-door vending, and then running shops in townships and business districts of bigger towns such as Johannesburg, Cape Town, and Durban.

One of the factors facilitating and giving momentum to Ethiopians’ migration to South Africa lies in the relatively liberal policies of both countries. Migration to South Africa has been enabled by a more liberal immigration policy post-Apartheid, at least until 2011. Meanwhile, a 1991 policy reform in Ethiopia has also protected people's constitutional right to movement, including international travels. Previously, Ethiopians needed an exit visa that highly constrained their movement.

Hadiya migration is also situated within the broader, historically shaped regional inequality between the Ethiopian “core North” and “peripheral South” that Hadiya is part of, which emerged in the process of state formation and is now evident in political representation and national wealth distribution. The long-term impact of the northern Ethiopian imperial rule (also called the Orthodox Christian empire) on the southern region, which lost its previous political and economic autonomy, contains an aspect of structural violence leading to many Southern Ethiopians developing self-doubt that impinges on their capacity to aspire for a better life.

Despite promises made by the 1974 revolution to redress ethnic inequalities and greater political inclusion of peoples of the periphery into national affairs, the Hadiya continued to occupy a marginal status throughout the *Derg* military rule from 1974–1991 (Feyissa 2022).

After the end of the Derg, the Ethiopian People's Revolutionary Democratic Front (EPRDF) and its ethnic federalism kindled yet another hope for the socio-economic transformation of the periphery. However, EPRDF's self-rule did not translate into the kind of socio-economic transformation the Hadiya expected. Meanwhile, the Hadiya National Democratic Organization's resistance against the EPRDF resulted in political persecution of its supporters (Tronvoll 2001), a further factor triggering youth migration to South Africa.

Moreover, the EPRDF's ethnic federalism worsened Hadiya's economic situation. One of Hadiya's coping strategies for the ever-growing population pressure on their land has long been internal labor migration to the coffee farms in southwestern Ethiopia or the sugar estates in the Awash Valley, particularly in Wenji (Negash 2017). Thousands of Hadiya migrants previously settled in Wenji, now under the Oromia regional state, had to go back to Hadiya as per the impositions of the new ethno-federal rule. Since Hadiya was already suffering from land shortages and high population density (one of the highest in Ethiopia), it could not absorb the return of internal migrants—nor were widespread employment opportunities available. This sparked a social crisis, which triggered additional migration.

Summarizing, there are a number of tangible factors that contributed to the onset and evolution of Hadiya migration to South Africa, including liberal migration policies in both origin and destination countries, historically shaped inequalities, political persecution of the Hadiya and economic drivers. In the following sections, we turn the spotlight onto the intangible and subjective factors shaping Hadiya's migration decision-making, specifically beliefs, imagination, social norms, and emotions and feelings.

### Beliefs and Prophecies Guiding Migration Decisions

Scholars have long overlooked the intersections of religious belief and human mobility. However, “for people who inhabit a religious tradition, every aspect of life may be connected to something beyond the measurable world, something that can be called ‘the sacred’” (Saunders, Snyder, and Fiddian-Qasmiyeh 2016, 2). Belief and faith could function as resources or triggers for migrants when deciding to migrate, have an effect on migrants’ commitment to enduring the hardship of the migration or taking risks, and make sense of life in the place of destination (Hagen-Zanker and Hennessey 2021).

Spiritual resources for the decision-making processes in Hadiya migration to South Africa include prophecies, prayers, and gospel songs with migration content, which are core features of evangelical Christianity. The spiritual dimension of Hadiya migration is indeed situated within this prophetic tradition and prayer economy.^4^

A major social event, which was key for the migration corridor's formative stage, is a prophecy delivered by Peter Youngren, a white Canadian pastor, who came to Hosanna in 2001. An estimated 500,000 people are said to have attended his Hosanna Friendship Festival in 2001, where he delivered the prophetic message that has shaped Hadiya migration to South Africa ever since: “I saw a vision of God opening a Southern route for the Hadiya through which people will go and bring back prosperity to Hosanna.^5^”

This was a message of hope, which sacralized and endorsed migration as a path showed by God. The Hadiya claim a collective agency for the prophecy, in that God answered Hadiya elders’ intense prayer to help them overcome the social and economic deprivations they were facing at the time of the prophecy. This prophecy was also perceived as an affirmation of God's favoring of the Hadiya as “committed” Christians, as elaborated by Feyissa (2022). Literature on charismatic Protestantism in Togo and Ghana (Piot 2010; Lauterbach 2017), also shows how personal and material success can be defined as a mark of devotion. This ideology of individual responsibility—which bears individuals responsible for their well-being and social mobility through the “correct” following of religious principles, is usually a feature of most branches of Protestantism, but it is also very present in the Hadiya's conceptualization of their own “successful” migration, both as a people and as individuals.

Interestingly, while the prophecy never mentioned South Africa as a specific destination, the Hadiya themselves interpreted the “Southern route” as leading to South Africa. This particular corridor looked profitable in economic terms, so intangible factors have been entangled with tangible ones right from the beginning. It was also not an entirely unknown destination, as a few Hadiya had already migrated to South Africa via Kenya in the late 1990s.

Belief shapes decision-making, instilling confidence without caution. Religious people indeed find moral strength and perseverance in their beliefs, which makes them arguably better placed to cope with the adversity experienced during migration. As indicated above, blessings are also used as a normative framework to make sense of the pursuit of material success. Although Pastor Peter Youngren declined to do so—arguing that blessing does not work on demand, many more Ethiopian pastors, mostly from southern Ethiopia, went to South Africa and blessed its land. The Hadiya retrospectively view the large numbers of their community members migrating to South Africa as an indication that the prophecy has been fulfilled. Additional signs that the prophecy is working include the success of Hadiya migrants despite multiple obstacles and the consequent economic transformation of Hadiya through remittances. Return migrants mentioned that they could not recognize the Hosanna they left behind, because it has changed so much including now having high-rising buildings and several banks. A common saying in Hosanna is in fact “whichever way you turn in Hosanna, there is South African money.”

Local spiritual entrepreneurs have proliferated, claiming the power to divine individuals’ future including whether to migrate or not, when, and how. They call themselves *miriit agelgayoch* (sent by God to convey His messages to specific individuals), although their detractors call them *festal agelgayoch* (amateur door-to-door spiritual service providers) and liken them with *festal hakim* (“plastic bag doctors” indicating amateur health professionals). Some of the *festal/mirit agelgayoch* are seen as predatory but even those skeptical of their service consider their prophecy and migration counseling largely accurate. People interested in migrating often seek these specific migration services from *festal/mirit agelgayoch* or from pastors, though advice is also still received spontaneously. A return migrant recounted how his decision to migrate to South Africa was a direct result of an unexpected “revelation” by a pastor during a Sunday service.

The idea of migrating to South Africa was suggested by my brother who went there earlier. He told me to come to South Africa, which I did with the money that he sent me. But why I decided to go to South Africa was not because my brother sent me money but rather only after I realized it was God's will. On a Sunday service a pastor came straight to me and put his hands on my head and prayed. He then mentioned that God wanted me to go to South Africa and that unlike other migrants nothing bad would happen to me throughout the journey. I had my doubts when my brother advised me to join him. You know, I was not as young as the other migrants, and I feared I might not be able to withstand the challenges. All my doubts disappeared after the revelation by the pastor who said, ‘your journey has already been made.’ (Fikiremariam, a returned migrant interviewed in Jajura, July 5, 2021)

Moreover, *festal/mirit agelgayoch* and pastors also indicate to the parents of prospective migrants which specific family member has better prospects of success, convincing them that it is worth investing in a particular person whose migration project is ordained by God, hence ensuring “value for money.” These material aspects of religion are not necessarily new and studies focusing on religion have been paying increasingly more attention to material components of faith, such as markers of success and/or the resource flows. This has been described as the “material turn” of social sciences (Hazard 2013).

For some migrants, spirituality has quite a functional role. One research participant pointed out that it is very common for people to display “too much spirituality” (*mamenafes*), that is to give a spiritual justification to a choice (migrating) that they had, at least subconsciously, already autonomously taken. This affirms a common behavioral pattern discussed in the literature as “confirmation bias,” namely the tendency to pay more attention and higher trust in information that implicitly confirms someone's preferences or decisions (Czaika and Vothknecht 2014).

Among contemporary Hadiya, fate, which is traditionally understood as fixed, is negotiable through prayer, as evangelical Christianity puts a higher premium on their transformational agency. When asked about why they decided to migrate to South Africa despite the risks involved in the journey, many prospective and return migrants mentioned they want(ed) to check their fate, saying “if I die during the journey or in South Africa, it is because I am meant to be.” They would add “who knows when and how I die anyway, even if I do not migrate?” Praying features as a badge of confidence that downplays the risks of migration, a belief that simultaneously shapes aspiration and builds migratory agency. Prospective migrants often respond “*atseliyebetalehu”* when asked what preparations they have made for the journey, meaning “I got my plan prayed upon.” In some prayers, pastors even include specific information about the risky aspects and/or parts of the migration journey (Feyissa 2022).

It is important to clarify that spirituality not only plays a significant role in relation to mobility but also to immobility. Immobility is involuntary for some but desirable for many others (see also Schewel 2020). In some instances, Hadiya prophets and pastors cum migration counselors advise prospective migrants to drop their plan to migrate. The following story sheds further light on how decision-making, including the decision to stay is shaped by a prophetic tradition:

There was a spiritual father called Aba Gole in Anlemmo, where I was born and grew up. He was a well-known religious leader who extensively traveled throughout Ethiopia. One day he visited my parents before I was conceived. He prayed and told my mother that she would have a baby boy and that boy would continue his legacy of preaching. So, I am a result of that prayer that shaped my purpose in life, serving God and His people. However, my mother's family who are Gurage (least connected to migration to South Africa) wanted to send me to South Africa and proposed to pay for my flight so that the life of the family will be changed. But I refused. Then in 2009, when I finished high school (my mother told me that) Aba Gole's sweat (was) in our family so I had to carry his legacy. I prayed the whole night and got my answer that said, ‘I will join theology college’. Thus, I ended up studying theology and I became a pastor, as prophesied by Aba Gole. (Pastor Samuel, Hosanna, 2021)

In this case, both tangible and intangible factors are important for decision-making, but it was interesting to see that, although the information and financial help offered by the brother were essential, it ultimately was the spiritual authority of the pastor that triggered and legitimized the decision to migrate. In other cases, people with little or no money and information set on the journey exactly because of the confidence generated by “having had their plan prayed upon.” All across the board, however, the relevance of spirituality is strongly related to the material allure of South Africa as one of the strongest economies in the continent. After all, the “evidence” of the fulfilled prophecy of 2001 has a strong material component.

In addition, Gospel songs are important aspects of belief that shape the decision-making processes of prospective Hadiya migrants. One is called *Chaltoto*, which in Hadiya language means “overcoming the challenges of the journey.” This originally referred to the spiritual sense of the term “journey to heaven” but is increasingly imbued with migration content. Some lyrics of the song say, “we will go, pass the hurdle, and inherit the kingdom regardless of the storm of the sea.” This is the most favored Gospel song played when prospective migrants and their planned journeys are blessed by a pastor, during farewell parties, and the most listened to during the journey. In all these occasions, the meaning decoded is, “you will make it to South Africa despite all the challenges.”

The spirituality of migration is also evident in the framing of the historically shaped regional inequality already mentioned. Migration to South Africa is understood as a means to renegotiate the inequality between the “core North” and the “peripheral South” which defines the Ethiopian polity, as the following commentary by a return migrant suggests:

How come that Amharas, Tigrayans and Oromos^6^ are not migrating to South Africa as much as the Hadiya and other Southerners do? Their oversight is not accidental. God has blinded them of this opportunity protecting it for us. Had they known about the opportunities in South Africa they would have taken it up as well. They are everywhere. Many Ethiopians in Europe, the United States and Canada are Amharas, Tigrayans and Oromos. They have money, knowledge, and wider social network. And yet we managed to make it to South Africa despite our apparent lack of skill and political networks. This is because God awakened us (*aberalin).* (Interviewed in Hosanna, 2021)

The word *aberalin* used here refers to a collective self, explaining that God has been engaging Hadiya as a people. In this sacred narrative, Hadiya migration to South Africa features as a quintessential spiritually animated future-making project, which at the same time guarantees tangible socioeconomic mobility.

However, the role of belief in migration decision-making has in recent years given way to other more secular imaginations, heralding a materialist turn in the Hadiya migration project. But still, we find Hadiya blaming themselves, not God, for migrations that went astray—evident in the rising difficulties during the journey (increasing death) and at the destination (homicide, xenophobic violence, robbery). In fact, Hadiya society is going through a reflexive moment occasioned by the feeling that God has given them “the migration gift” but they are not making the best out of it. A return migrant thus surmised:

We are yet to learn how to work together, not just helping each other to migrate and eat together. Failing to do so, we have turned a blessing into a curse, a promised land into death land. (Interviewed in Fonqo, 2021)

Participants particularly refer to the rising homicide rate among Ethiopians in South Africa and the moral/spiritual decay connected to it—that sharply contrasts with the Hadiya's self-understanding as devout Christians. In fact, many return migrants acknowledge most of the violence in migrant communities in South Africa is committed by migrants themselves, though some are seen as having links with South African criminal groups.

As noted by a return migrant, this “moral decay” that underpins migration processes involves what can be called “spiritual laundering”:

Those who kill and engage in corrupt practices give offers to a church and feel absolved of their sins. Parents are now less happy and cooperative in sending their children to South Africa. On the face of it, the previous societal consensus on migration has unraveled. There is now a feeling that unless Hadiya use the gift of migration in a responsible manner, a *bereket* (blessing) could be turned into a *merigemt* (curse). (Interviewed in Hosanna, 2022)

### The Evolution of Imaginaries

Imagination is one aspect of decision-making that concerns a variety of mental processes, such as contemplating or visualizing a destination and/or someone's life in a different place at some point in the future (Hagen-Zanker, Mazzilli, and Hennessey 2023). Such processes have been described, among others, as “mental journeys” and “imagined mobilities” (Cangià and Zittoun 2020), “mental simulation” and “mental time travel” (Kyle and Koikkalainen 2011). This multiplicity of definitions reflects the complexity of the concept of “imagination” (Hagen-Zanker, Mazzilli, and Hennessey 2023), which can be used to better understand how rich and multifaceted migration decision-making processes are.

For the Hadiya, Pastor Peter's prophecy initially generated a particular spatial imaginary of South Africa as Hadiya's promised land. This is further reinforced by South Africa being perceived as a Christian, or, at least, pluralistic country, in contrast to other migration destinations and therefore a suitable place for Hadiya where to establish a new life. The importance of a place's religious identity can be seen from a parallel between Ethiopian migration to South Africa and to the Gulf countries. Ethiopian migration to the Gulf started around the same time as that to South Africa, but Hadiya's response to this migration corridor was lukewarm at best. Although some Hadiya girls migrated to the Gulf despite being discouraged by their parents, the attractiveness of this destination could not compete with South Africa, at least in the spiritual and religious imaginary. South Africa is indeed regarded as a sacred destination. Belloni (2022) put forward the concept of “cosmologies of destination,” the hierarchical representations of geographic imaginaries grounded on several elements such as moral righteousness/decay, according to which some locations are imagined as “good destinations” while others are not.

The foundational prophecy and its resulting migration flows have not only generated a spatial imaginary of South Africa as a destination but also of imagined lifestyles. Moves to another place are the ultimate opportunity to reinvent oneself and, during the decision-making process, people may be imagining what a different lifestyle or altered identity could look like (Hagen-Zanker and Hennessey 2021). For the Hadiya, both as individuals and a community as a whole, coming from a disadvantaged, marginalized region with few economic or life opportunities, migration was an opportunity to refashion their role from being on Ethiopia's periphery to join a “global community,” often starting via an initial rural–urban move to Hosanna. This chimes with other studies focusing on members of marginalized communities who imagine migration as an opportunity to enable a “modern,” global lifestyle (Raitapuro and Bal 2016; Brown, Scrase, and Ganguly-Scrase 2017).

As before, these imaginations are tied to tangible factors, such as the mansions built by successful Hadiya migrants and/or their investments in Hosanna. Habtamu's story illustrates both the imagined lifestyles and strong desire to be a part of a more modern society, fueled by jealousy, and competitiveness:

I had a government job before I migrated to South Africa. I decided to migrate mainly because I could not bear the sense of loss, having seen people from our village who went to South Africa, came back, and bought a FSR car. […] At that time many Hadiya migrants bought FSRs and everybody I knew driving an FSR car would be a migrant. And I thought the only means of catching up, and catching up fast, would be going to South Africa. (Interviewed in Jajura, 2021)

These imaginaries are fed by multiple sources. The evangelical church plays quite a strong role in this, acting as migration advisors as discussed above. Moreover, word of mouth and rumors of Hadiya's success in South Africa and the videos shared on social media fuel these imaginaries (see also Kyle and Koikkalainen 2011; May 2004 among others). Whereas migrants nowadays mostly communicate and obtain information—and, crucially, inspiration—through apps such as imo and Facebook, in the early days imaginations were fueled by videos recorded by Ethiopian migrants in South Africa during special occasions such as weddings. Pioneer migrants would send their wedding videos back home, which family members and friends or acquaintances would watch at video shops for a fee. Over time, Hadiya migrants have increasingly adopted the flamboyant wedding ceremonies practised in South Africa, thereby sending a clear message of economic success.

These imaginations focus on the successful migrants, whose businesses are prospering, those who made the journey successfully, rather than the many more Hadiyas in South Africa who are struggling to turn a profit or who are the subject of violence and xenophobia. This is a common cognitive process of decision-making described as “tunnel vision” (Czaika and Vothknecht 2014), similar to but not quite the same as confirmation bias. Tunnel vision essentially blocks out information that does not fit a predetermined idea or imagination. Preachers, prayers, and prophecies further reinforce this one-sided picture, making the idea of migration even more attractive and almost inevitable for many.

In fact, migration has in some ways transformed imaginations of what constitutes a successful and socially accepted social mobility pathway. In earlier generations, education and public service were seen as the not only accepted but also desired pathways to social mobility, migration has both displaced and devalued this. This was shown above in the story of Habtamu, who left his public sector job to migrate to South Africa. The story of Asham, a PhD candidate at Addis Ababa University, below, demonstrates that even when education results in positive economic outcomes, being highly educated is no longer a socially desired life for the Hadiya:

In my time (1990s), it was education which was sought after and passing the national exam was such a big thing. My father invested in his children's education. No one from our family migrated to South Africa. It was only two of us—myself and a friend—who got the highest grade in Hosanna and joined university. The important social distinction then was between *ye chane* (those who have a university degree) and *yal chane* (those who don’t). That was why, when the news spread that I passed the national examination, neighbors, and some from far brought to my family 20 coffee pots to honor my success, as per the Hadiya tradition. Years after, I was no longer the socially attractive one. Less intelligent people who made it to South Africa became the new hero. People now mock me saying ‘before and after graduating from the university your father has lived in a mud house with a thorn fence, but migrant families live in fancy houses!’ They also mock me that the reason why I married a non-Hadiya girl is because I couldn’t afford the bride wealth or simply because a Hadiya girl will no longer consider a university degree as a relevant factor in partner selection. Even the fact that I will soon be a doctor is not helpful. I am a PhD candidate at Addis Ababa University. Elsewhere in Ethiopia, people with a PhD degree are still highly respected. They are even called ‘doctor’, as if this is a personal name. The title carries a lot of social weight. My father used to be very proud that all his children were educated. (…) He still feels very proud of us; but he has come under strong social pressure. Once he asked me to send him money for a public holiday, but he said, ‘please make sure that you will not send the money through the bank but send it through people coming to Hosanna’. This was because he knew that what I normally send family would not be more than 2,000 birr. He would feel embarrassed to take out such a ‘small’ amount of money from the bank in front of the public in a situation where migrant families bring plastic bags to collect remittances. (Interviewed in Jajura, 2021)

Migration thus fulfills imagined identities and lifestyles granting prosperity, social acceptability, and advancement within the Hadiya society and Ethiopia.

### Norms in an Egalitarian Yet Competitive Society

Hadiya society is, on the one hand, guided by the idea of equality, according to which people are born equal and should strive to maintain a balance with their peers throughout the course of their life (Feyissa 2022). Contrary to what happens in other African societies, in which individual accumulation is discouraged to suppress social inequality through a system of redistribution (see for instance Lewis Wall 1976 for accumulation and redistribution among traditional Anuak society), Hadiya people feel compelled to continuously catch up with each other's successes: in this way individuals can level up with others and everybody's goals keep moving forward. The importance of someone's relative social status within their own reference group—first identified in the 1980s under the New Economics of Labor Migration—is well known in the literature as a driver of migration. This approach acknowledges that it is not only income differentials between countries of origin and destination which cause migration, but also other less tangible factors such as risk management or relative income/social status within a community (Stark 1991; Massey et al. 1993).

The surprising flip side of equality is fierce competition, which in Hadiya society can be seen particularly clearly among the youth. With the opening of the migration corridor to South Africa, competition among Hadiya shifted from the realm of education to a different level. Those who migrated to South Africa have been able to buy commodities such as a car or a house and have the means to give conspicuous charity offers to their local church. This has made them respected members of society, and their new status has triggered the migration of more and more individuals, especially young ones.

For Hadiya, migration has now become part of the natural order of things, in which both long-standing cultural norms of society and new trends converge. Concerning cultural norms, Hadiya perceives migration to South Africa as one—perhaps the ultimate—expression of *darifirma*. While the literal meaning of this word is “to stroll,” *darifirma* commonly symbolizes the physical mobility that Hadiya has experienced and enjoyed since the beginning of time. As Feyissa (2022) points out, in this context migration is not only seen as such, but also as an expression of the human disposition to move, thus perfectly natural (p. 37). The egalitarian but competitive nature of the Hadiya society, together with the guiding principles of *darifirma*, provided a fertile cultural ground for migration. Its influence is pervasive in that not only young people feel they have to migrate to be someone, but also their parents enjoy the indirect prestige coming from the stories about children abroad.

The story of Dr Wamicho and his father is key to understand both the connection between migration and high social status and its flip side, that is the relative deprivation perceived by those who do not have migrants in the family. Dr Wamicho is an academic and a member of the senior management of Wachemo University. His father comes from a traditionally rich family and owns over a hundred hectares of land—a lot for Hadiya standards, where a family owns on average 0.5 hectares. Notwithstanding Dr Wamicho's academic achievement, his father felt deprived because there is no one in their family who migrated to South Africa:

My father once said, ‘when I hear all kinds of stories about South Africa and how children send money to their parents and bring them to towns, it feels to me as if I did not have any children.’ I asked him why he needs his children to migrate, as he is already rich. What we need is not someone to migrate but someone to work in my family's fields. But he said, ‘I do not have any story to tell my friends whose children are in South Africa. You guys have made me story-less.’ I then decided to build him a house in Hadero town. He was happy but still really needed one of his children to migrate to South Africa. He tried with his daughters: I intercepted a plan to send my elder sister as a wife to a migrant in South Africa. But resisting to the second attempt became too much. My father sent an elder who threatened to curse me if I did not agree to his plan. So, I complied and soon my sister will fly to South Africa. Her husband will pay all the expenses, for a total of 1.2 million birr. The plan is that she will take a safer route; a flight from Addis Ababa to Mozambique and then cross into South Africa.^7^ This is how important South Africa has become in the local status system. (Interviewed in Hosanna, 2022)

### Emulative Jealousy

Egalitarianism and competition have been so far discussed as social norms comprehending an entire community but, in addition to them, also personal emotions and feelings influence Hadiya's migration decision-making. Jealousy and social pressure are known to influence migration behavior (Hagen-Zanker and Hennessey 2021), especially in contexts where there is a culture of migration and/or where the expectations that certain categories of people migrate are strong. Studies conducted in Cambodia (Bylander 2015), Ethiopia (Belloni 2019), Gambia (Gaibazzi 2010), and Tunisia (Zagaria 2019) all identified social and peer pressure to migrate. In some cases, such as the one described by Bylander (2015), even those who are personally not interested in migrating feel compelled to do so by the disrespect of their peers, who look down on them for not following “the norm.” Similarly, several studies have documented how the shame of not having achieved “success” through migration either pushes individuals to migrate onward (Dako-Gyeke 2016; Hernandez-Carretero 2016) or discourages them from returning (Staniforth 2014; Chan 2018).

However, in many cases—such as the one we examine in this article—migration is more of an aspirational project than a product of social pressure. For instance, Gaibazzi's (2010) study of Soninke young men in the Gambia discusses how the prestige acquired by those who migrate generates “emulative jealousy” in their peers who have been left behind, pushing them to migrate as well. The counterpart of jealousy is frustration, which arises from the living situation of those who have not (yet) migrated (van Bemmel 2020). In the context of a highly egalitarian society such as the Hadiya, those (still) in Ethiopia feel frustrated because they are left behind and long for success, which they feel they deserve as much as their peers.

Social pressure, emulative jealousy, and frustration are reportedly much more common among young men than among young women. Ideas of masculinity and of rites of passage to adulthood (see Zagaria 2019) are undoubtedly at the basis of this, although research has demonstrated that these feelings are by no means a male prerogative (Grabska 2020). In this article, we focus on young men's perspective, as Hadiya migration to South Africa is very much gendered, with boys migrating on their own or with their peers and girls mainly being “called to” South Africa from a fiancé or husband—thus the data collection behind this article represents their perspective much more in detail.

The consolidation of the migratory corridor from Ethiopia to South Africa over the past 20 years, together with the ever-more certain prospects of success in South Africa, have tinged emulative jealousy and frustration with a sense of urgency. It is hard to establish with absolute certainty whether this urgency is a symptom of the individualized character that Hadiya migration has assumed over the years, which places the entire responsibility (but also merit) of success on the migrant, or of the “urge to catch up” (Feyissa, Zeleke, and Gebresenbet 2023) typical of the egalitarian and competitive Hadiya society. We suggest both influence it. Dula, one participant, described how a decade ago parents would impose on their children—especially irresponsible or lazy ones—migration as a disciplinary measure through which they were challenged to “become somebody,”

(Migration to South Africa) was imposed on me by my parents. I was the naughtiest of all their children. I started smoking and drinking at an early age. I even joined gang groups in Hosanna and participated in hanging. My father tried many things in vain. He finally thought of sending me to South Africa. He said to me ‘go to South Africa and be somebody’. He did not mean this in the sense of go there and make money but rather he believed in the transformational power of migration. Many people who do not do anything would become hard workers in South Africa and come back not only with money but also as a better, responsible persons. South Africa proved my father right. I migrated reluctantly but now I am a successful businessman who owns a house, plots of land, shops, and trucks. Above all, my parents are now happy with me. (Interviewed in Shashogo 2021)

Nowadays this dynamic has reversed. A resident of Hosanna who made the conscious decision to stay and advance in the educational ladder shared the following account of the strong desire to migrate among youth:

Some of the boys carry knives with them and they blackmail their parents to send them to South Africa. Children go to school only until they turn 15, when they feel they have come of age, and it is their moment to go to South Africa. Even 10-years-old boys are putting pressure on their parents either to send them to South Africa or buy them a motorcycle or *bajaj* (tuktuk). Other than to buy land, most of remittances are now used to buy motorcycles. In fact, everyone wants to have one, especially the youth. Boxer motorcycle is considered almost as a domestic animal, as everyone wants to have one. The youth left behind are not interested in farming: they rather charge 50–60 birr to transport farmers to towns with their motorcycles and save the money they make so that they pay for their migration. (Interview with Dr Torsido, Hosanna, July 2021)

This has become the new normal and parents are just complying with it—migration is now factored into families’ life plans. This account also explains well how migration is the end goal for the youth, an intrinsic goal in itself, which they reach either when their parents send them to South Africa or once they have saved enough money using their motorcycle as a transportation service.

## Conclusions

Our article presented the case of Hadiya migration to South Africa to introduce a conceptual framework in which we put intangible factors at the core of migration decision-making. However, we do not reject or downplay the importance of tangible factors, rather acknowledging that intangible and tangible continuously inform each other. We built on the literature on migration decision-making, which has been expanding over the past decades, leaving behind the old dichotomy between the functionalist and historical-structuralist approaches—both too simplistic each for a different reason. Adopting Hagen-Zanker, Mazzilli, and Hennessey’s (2023) classification of subjective and intangible factors into the four main categories of imagination, personality traits, emotions and feelings, and beliefs and values (including societal norms), we showed that spirituality, egalitarian social norms, and feelings of admiration and emulative jealousy lie at the core of Hadiya migration to South Africa. The decision to migrate to South Africa is shaped by a foundational prophecy which in 2001 sacralized the Ethiopia-South Africa corridor, depicting South Africa as Hadiya's promised land. Together with prophecy, we showed that prayers and gospel songs with migration content are used strategically to “secure” a positive outcome for the migration journey and infuse “confidence without caution.” This confidence in turn grounds on Hadiya's egalitarian societal norms, which generate feelings of admiration but also emulative jealousy in those who have not yet left for South Africa.

The connotation of South Africa as a promised land is supported and reinforced by material considerations. South Africa is one of the wealthiest economies of the African continent, where more relaxed laws in comparison to Ethiopia allow for relative ease in business establishment. In addition to this vision of the destination, Hadiya see themselves as the “chosen” people, which once again grounds on material aspects as well as intangible ones. The political, social, and economic regional inequality experienced by the Hadiya within Ethiopia is conceptualized through a spiritual scheme of interpretation where migration features as a divine balancing act, helping the Hadiya to renegotiate a fair distribution of public goods and status between them and the members of Ethiopia's dominant groups.

However, the relevance and significance of migration keep changing over time. Nowadays, migration has displaced other means of socioeconomic mobility. Emerging as a collective project of salvation for the Hadiya people, migration to South Africa is slowly transforming into a signifier of individual worth and a promise of material and personal success. Its influence is pervasive in that not only young people feel they must migrate to be someone, but also their parents enjoy the indirect prestige coming from the stories about sons and daughters abroad. The youth have gone one step further, making a case for migration at any cost, imbued with a sense of urgency, while parents have become more cautious because of increasing risks not only during the journey but also at destinations. This material turn in the Hadiya migration project and its increasing individualization is accompanied by a reflexive moment among the Hadiya public, pondering whether what was given to them as a divine gift is rapidly turning into a curse. That more and more Hadiya parents are now discouraging their children from migrating to South Africa despite the apparent material bonanza shows yet again how the tangible is reinforced, critiqued, and made sense of through intangible factors operating not only at the individual but also collective level.

Further research should expand onto other geographical contexts, which will allow us to reveal the breadth of intangible factors that can constitute core drivers of migration decision-making and the manifold ways in which tangible and intangible factors interact. Enriching the pool of studies grounding on the conceptual framing proposed here can ensure their robustness and contribute to the understanding of many aspects of migration decision-making that are still hard to grasp—ultimately doing justice to such a diverse and rich human experience as migration.

## Notes

1 See de Haas, 2021 for a more detailed discussion on the evolution, merits, and concerns of these two approaches.

2 The “Five Factors” model developed by McCrae and Costa in 1989 established the traits of (1) openness to new experiences, (2) conscientiousness, (3) extraversion, (4) agreeableness, and (5) emotional stability as the pillars of personality classification.

3 Specifically, 60 percent of the overall 100 research participants strongly linked migration with spirituality; 30 percent connected them loosely, and 10 percent did not make any reference to subjective factors, instead only referring to economic and political reasons behind their decision to migrate.

4 The term “prayer economy” has been coined by one of the authors and inspired by “moral economy.” Prayer economy is transactional but goes beyond that, including feelings and beliefs.

5 Hosanna is the capital city of the Hadiya administrative zone.

6 These are all populations living in the “core” regions of Ethiopia.

7 At the time of writing, Wamicho’s sister had already migrated to South Africa.
